# The Outcomes of Selective Nerve Root Block for Disc Induced Lumbar Radiculopathy

**DOI:** 10.5704/MOJ.1511.002

**Published:** 2015-11

**Authors:** K Arun-Kumar, S Jayaprasad, K Senthil, H Lohith, KV Jayaprakash

**Affiliations:** Department of Orthopaedics, Melmaruvathur Adhiparasakthi Institute of Medical Sciences and Research, Melmaruvathur, India

**Keywords:** Selective Nerve Root Block, Disc prolapse, Radicular Pain, Lumbar Radiculopathy

## Abstract

Introduction : Use of Selective Nerve Root Block (SNRB) in lumbar region to relieve radicular pain along the course of a particular nerve is being practiced in the recent past. We observed and tabulated therapeutic outcomes of this procedure in management of radiculopathy induced by a prolapsed disc affecting a particular lumbar nerve root.

Methods : 40 patients with various grades of disc prolapse affecting a particular lumbar nerve root presenting with chronic radicular pain were identified irrespective of age and sex. All were injected with a combination of 40 mg of Methylprednisolone based suspension with local anaesthetic over the affected nerve root and results were analyzed.

Results : Those graded mild had 4.3 months relief and those graded moderate had 2.5 months relief. Those with severe disc prolapse had no relief except for the immediate postprocedural relief. Only 20% patients had relief up to 6 months.

Conclusion : Effect of SNRB is typically short acting in majority of patients and recurrence is expected. It creates a window period with reduced pain but of varied intervals depending on the pathology. It did not alter the prognosis in those with severe disease where surgery is well indicated.

Level of Evidence – Level 4

## Introduction

Lumbar radiculopathy can be defined as pain from lower back radiating until the leg or further beyond along the course of a particular lumbar nerve. Selective Nerve Root Block (SNRB) is practiced as a part of the management of radicular pain due to a particular affected nerve root in both cervical and lumbar regions^1-3^. Although its specificity as a diagnostic tool is said to be low, Therapeutic efficacy needs to be debated^4, 5^. It is used invariably for those with or without significant surgical spinal lesions^6^. Mechanical lesions include various stages of disc prolapse, ligamentum flavum hypertrophy, facet hypertrophy and degenerative osteophytes causing foraminal stenosis, all leading to nerve root irritation^7^. Inflammatory response to exposed nucleus pulposus is also said to contribute to the nerve root pain^7^. The principle behind this technique is to reduce inflammation of the nerve root by injecting a steroid and thus reducing the intensity of pain. But the actual pathology causing the nerve root irritation remains and hence recurrence is expected. Our aim is to study the prognosis after single dose of SNRB over affected lumbar nerve roots and find out whether a window period of reduced pain be achieved before proceeding to next line of management.

## Materials and Methods

Selection criteria of our patients were irrespective of age and sex. Those patients with complaints of lumbar radiculopathy for more than 3 months demonstrating a positive unilateral Straight Leg Raising test (SLRT) within 30-60 degrees were selected. MRI was done in all patients as a standard protocol to look for mechanical lesions. Only those patients with intervertebral disc lesions affecting a particular lumbar nerve root were selected for the study. Those with more of back pain component than radiating pain were excluded. Those with bilateral symptoms, multiple nerve root involvement and neurological weakness were excluded. We used the MSU Classification for herniated lumbar discs on MRI to grade our patients (Table I)^8^.

**Fig. 1:**
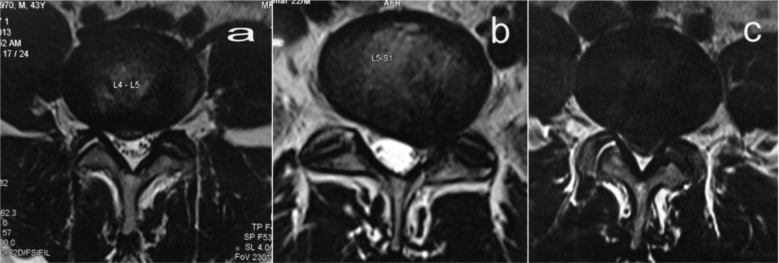
Figure 1a : Mild disc prolapse. Figure 1b : Moderate disc prolapse. Figure 1c : Severe disc prolapse.

Our study group consisted of 40 patients, 9 male and 31 female. Age of our patients was between 23 – 61 years with mean age being 42.6 years. 23 patients had right sided radiculopathy and 17 patients had left sided radiculopathy. All patients were given the Roland Morris Disability questionnaire (RMDQ) for back pain and their score was recorded before the procedure^9^. Numeric rating scale (NRS) for pain was used to grade pre procedure pain on doing SLR^10^. Radiological lesion in 15 patients was type 1B prolapse (Fig 1a) and in 18 patients was either 2B or 2AB prolapse (fig 1b). The remaining 7 patients had a 3B or 3AB prolapse (Fig 1c) where surgery is very well indicated but were not willing to proceed with immediate surgery. Our sample did not contain patients of all 10 types described in the classification. For discussion purpose we describe those with type 1B prolapse as mild, those with 2B or 2AB prolapse as moderate and those with 3B or 3AB prolapse as severe (Fig 1). Procedure took not more than 15 minutes.

All selected patients had previously undergone a course of conservative management with rest and physiotherapy which did not relieve their symptoms. 32 patients had L4-L5 disc prolapse which usually affects the L5 nerve root that traverses at that level and exits below the L5 pedicle. 8 patients had L5-S1 disc prolapse that usually affects the S1 nerve root that exits in the first dorsal sacral foramina. Our sample did not have patients with pathology at any other level except for L4-L5 and L5-S1 and hence our target nerve roots were L5 and S1. None of our patients had a L4-L5 far lateral disc prolapse affecting the L4 root and none had a L5-S1 far lateral disc prolapse affecting the L5 root.

**Fig. 2:**
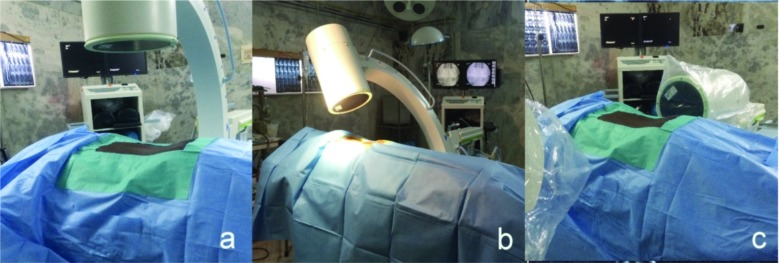
Figure 2a : C-Arm positioned for an anteroposterior (AP) view to target L5. Figure 2b : C- Arm in 30 degree caudal tilt for obtaining true AP view of sacrum to target S1. Figure 2c : C-Arm positioned for lateral view.

Our procedure to target L5 and S1 roots differs. It is always done under C-Arm guidance but the position of C-arm and direction of exposure varies (Fig 2). Patient is made to lie down prone and their lumbosacral spine region is prepared and draped. C-Arm is positioned for an antero-posterior (AP) view of the lumbosacral junction (Fig 2a). Targeting the affected L5 nerve root requires identification of L5 pedicle on that side in an AP C-Arm image. The L5 nerve usually exits below and close to the L5 pedicle. Adequate local anaesthetic was infiltrated under the skin 3 – 4 cms lateral to the inferior border of L5 pedicle where we usually enter. An 18 gauge spinal needle was introduced and directed to a point few millimeters below and lateral to the L5 pedicle where the nerve is usually found. If bony resistance is felt then the needle is walked over the bone to reach the desired point. Targeting S1 nerve root requires the C- Arm to be tilted perpendicular to the sacrum so that there is a clear view of the first dorsal sacral foramina (Fig 2b). Here the needle is directed into the first dorsal foramina where S1 exits.

**Fig. 3:**
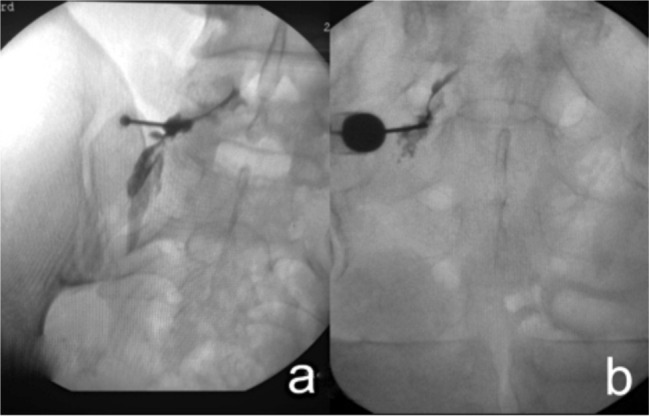
Figure 3a – Spread of dye along L5 root Figure 3b – Spread of dye along S1 root

Patient is cautioned about the paresthesia which will be felt along the course of their radicular pain when the needle touches the nerve and due to this reason we were very cautious not to handle the needle vigorously. This is to prevent injury to the nerve caused by the needle. A lateral view was taken occasionally to confirm position of the needle in circumstances when there was difficulty in eliciting paresthesia (Fig 2c). Once paresthesia is elicited, needle is slightly withdrawn and 0.5 ml of Iohexol (an iodine based radiopaque dye) is injected to confirm the position of needle^11^. Various patterns of contrast material distribution around the nerve were seen as shown in literature (Fig 3)^10^. Then a combination of 40 mg of Methyl prednisolone based suspension with local anaesthetic was injected over the affected nerve root. Post procedure parasthesia due to local anaesthetic effect is expected.

Numeric rating of pain using NRS on doing SLRT was used to analyze immediate effect of this procedure. Most of the patients were discharged on the day of procedure and were asked to be in rest for the first 2 days. They were asked to review after 2 days if symptoms remain unchanged or after a week if improvement is felt. Patients were given the Roland Morris Disability questionnaire for back pain and their score was recorded every week in the first month after procedure and every month thereafter. Those with unchanged symptoms and recurrence went for a subsequent decompression surgery and remaining patients were warned about recurrence of symptoms.

## Results

Mean numeric rating of pain using NRS before the procedure on doing straight leg raising on the affected side was 8. Mean pre procedural Roland Morris Disability questionnaire score was 23. 32 patients (80%) had L4 L5 intervertebral disc prolapse in whom L5 nerve root was targeted. 8 patients (20%) had L5 S1 disc prolapse in whom S1 nerve was targeted. Mean numeric rating of pain using NRS immediately after the procedure on doing straight leg raising on the affected side was reduced to 4 which is due to the local anaesthetic effect. NRS assessment on doing SLR was done on 2nd day after the procedure for 7 patients who returned with similar pain. It was found to be the same as pre procedural status or one point less. They were given reassurance and were asked to wait until one week is complete for RMDQ score. All patients were reviewed after one week and were given the Roland Morris Disability questionnaire for back pain and their score was noted. Mean overall RMDQ score at 1 week was 10.35 which denote improvement. Those with full recurrence in subsequent follow ups having RMDQ scores more than 20 were excluded from our sample to proceed to next line of management which is not discussed in this article.

Even though overall RMDQ score denotes improvement by 1 week, 7 patients had RMDQ Score of 20 or more. These patients were the ones considered severe in whom surgery was indicated but were not willing to proceed with immediate surgery. All these patients had full recurrence after 1st week with RMDQ score of 22 or more. They were explained about their poor outcome and all of them opted for a surgical decompression. On excluding those 7 patients with recurrence our sample reduced to 33 patients (82.5%) with a mean RMDQ score of 8.1 by 2 weeks and 6.2 by 3 weeks. Review at one month had 4 patients with RMDQ scores more than 20. Hence they were excluded for further management reducing our sample size to 29 patients (72.5%). The mean RMDQ score of the reduced sample was 7.8 by 1 month.

**Table I:** Grading of herniated discs based on MRI

	Table I - Grading	
No. Of Patients	Grade	Type Of Prolapse Based On Msu Classification
15	Mild	Type 1b Prolapse
18	Moderate	Type 2b/2ab Prolapse
7	Severe	Type 3b/3ab Prolapse

**Table II:** Change in RMDQ score with follow up

	Table II – Analysis			
Follow Up		No Of Patients With Relief	Percentage	Mean RMDQ Score
2 Weeks		33	82.5	8.1
3 Weeks		33	82.5	6.2
1 Month		29	72.5	7.9
2 Months		19	47.5	6.8
3 Months		13	32.5	9
4 Months		8	20	8.4
5 Months		8	20	11.8
6 Months		8	20	15

**Table III:** Outcome grading based on duration of pain relief

	Table III – Results Based On Pathology
Grade	Mean Duration Of Relief
Mild	4.3 Months
Moderate	2.5 Months
Severe	Only Immediate Post Procedural Relief

Next follow up was by 2 months which had 10 patients with RMDQ scores more than 20. They were excluded and our group reduced to 19 patients (47.5%) with mean RMDQ score of 6.8. Six patients had recurrence at 3 months reducing our group to 13 (32.5%). Mean RMDQ score of the remaining 13 was 9. By 4 months, 5 more patients had RMDQ score more than 20. Hence our group reduced to 8 patients (20%) with mean 4 month RMDQ score of 8.4. These 8 patients were seen at 5 months with mean RMDQ Score of 11.8 and at 6 months with mean RMDQ score of 15. Patients with non-surgical radiological lesions who had symptoms after 6 months were given choice of a 2nd dose SNRB and all of them opted. They were explained about our results and prognosis.

Analyzing results showed that 82.5% patients had improvement by 2 weeks which reduced to 72.5% by 1 month (Table II). Sequential follow up revealed constant decrease in the percentage of patients with relief. Only 47.5% of patients had relief by 2 months which reduced to 32.5% by 3 months and to 20% by 4 months. Finally only 20 percent patients remained at 6 months progressing slowly back to pre-procedural status. The last 20% patients were those with mild protruding disc towards one side. According to our description based on MSU Classification, those graded mild had 4.3 months relief and those graded moderate had 2.5 months relief. Those with severe disc prolapse in whom surgery is indicated had no relief except for the immediate post-procedural relief (Table III).

## Discussion

Lumbar radiculopathy is a very common condition seen in our orthopaedic clinic. We see an increased incidence in patients presenting with this condition. Conservative management in these patients is highly unpredictable. Most patients do not accept surgery in first place and there are circumstances where we feel surgery is not yet indicated. Such patients require something that will relieve their pain at least for a short duration. SNRB plays an important therapeutic role in these patients. As the actual pathology causing the nerve root irritation remains, prognosis in these patients varies.

Many authors have used methyl prednisolone based preparations for this purpose^12^. Triamcinolone and betamethasone based preparations are also in use^13^. Easy availability in our institute made us to choose a methyl prednisolone based aqueous suspension for this purpose. For obtaining therapeutic effect, we used 40 mg of steroid for one SNRB. To be more precise, we always looked for paresthesia when the needle touches the nerve root and then withdrew the needle slightly before injecting the dye. Care is taken not to handle the needle vigorously when around the nerve root as we expect a mere touch with the root. Most authors say that this should not be done to prevent needle induced complications but we did not have any such complications^11^, 14.

We used the numeric pain rating scale for pre procedural and post procedural assessment of pain^10^. The pain rating can be obtained by asking the patient to choose a number between 0 to 10 with 0 being no pain and 10 being severe pain. But our main aim was to concentrate on the functional outcome and hence we used the Roland Morris Disability Questionnaire^15^. This method is useful in circumstances when the patient is not able to come for follow up. It has a set of 24 questions that is asked to the patient mainly on functional aspects. Patients just need to reply yes or no for this questions hence a telephonic conversation with the patients is enough to collect data. The higher the number of Yes, the severe is the problem. Clinical improvement over time can be graded based on the analysis of serial questionnaire score^15^.

As noted by few other authors, early response did not predict the effect after 2 weeks^11^. There were patients with severe pain during first follow up who gradually improved and there were also patients with good relief gradually worsen. Those with severe disc prolapse who were not willing for surgery neither responded nor gained any interval period with reduced pain except for the immediate post procedural relief. This immediate relief can be considered as a diagnostic tool to confirm that the blocked root is the affected one that needs to be decompressed. It correlates with the amount of relief the patient will have if that particular nerve root is decompressed surgically. Those with mild and moderate prolapse showed similar results gaining them an interval period with reduced pain which allowed most of our patients to think about next line of management if their pain comes back.

## Conclusion

The effect is typically short acting in majority of patients yet it gains a valuable interval period of reduced pain in those patients with mild and moderate pathology. This procedure can be effectively used as an intermediate procedure before going for surgery in those patients with inconclusive radiological indication for surgery. It does not alter the prognosis in those with severe disease where surgery is well indicated.
